# Familial Mediterranean fever without *MEFV* mutations: a case–control study

**DOI:** 10.1186/s13023-015-0252-7

**Published:** 2015-03-25

**Authors:** Ilan Ben-Zvi, Corinne Herskovizh, Olga Kukuy, Yonatan Kassel, Chagai Grossman, Avi Livneh

**Affiliations:** Department of Medicine F, Sheba Medical Center, Tel Hashomer, Ramat Gan, Israel; The Rheumatology unit, Sheba Medical Center, Tel Hashomer, Ramat Gan, Israel; Heller Institute of Medical research, Sheba Medical Center, Tel Hashomer, Ramat Gan, Israel; Sackler School of Medicine, Tel Aviv University, Tel Aviv, Israel; The Dr. Pinchas Borenstein Talpiot Medical Leadership Program, Chaim Sheba Medical Center, Tel-Hashomer, Ramat Gan, Israel; Institute of Nephrology and Hypertension, Sheba Medical Center, Tel Hashomer, Ramat Gan, Israel

**Keywords:** Familial Mediterranean fever, FMF, *MEFV*, Mutations, Phenotype

## Abstract

**Background:**

Although familial Mediterranean fever (FMF) was originally defined as an autosomal recessive disorder, approximately 10–20% of FMF patients do not carry any FMF gene (*MEFV*) mutations. Fine phenotype characterization may facilitate the elucidation of the genetic background of the so called “FMF without *MEFV* mutations”. In this study we clinically and demographically characterize this subset.

**Methods:**

*MEFV* mutation-negative FMF and control patients were recruited randomly from a cohort followed in a dedicated FMF clinic. The control subjects comprised 2 groups: 1. typical population of FMF, consisting of genetically heterogeneous patients manifesting the classical spectrum of FMF phenotype and 2. a severe phenotype of FMF, consisting of FMF patients homozygous for the p.M694V mutation.

**Results:**

Forty-seven genetic-negative, 60 genetically heterogeneous and 57 p.M694V homozygous FMF patients were enrolled to the study. *MEFV*-mutation negative FMF patients showed a phenotype closely resembling that of the other 2 populations. It differed however from the p.M694V homozygous subset by its milder severity (using Mor et al. scoring method), as determined by the lower proportion of patients with chest and erysipelas like attacks, lower frequency of some of the chronic manifestations, lower colchicine dose and older age of disease onset.

**Conclusions:**

*MEFV* mutation-negative FMF by virtue of its classical FMF phenotype is probably associated with a genetic defect upstream or downstream to *MEFV* related metabolic pathway.

## Background

Familial Mediterranean fever (FMF) is an autosomal, recessively inherited autoinflammatory disease, associated with the MEditerranean FeVer gene (*MEFV*) and characterized by recurrent episodes of fever and serositis, mostly peritonitis, but also pleuritis, pericarditis and synovitis [1,2]. The *MEFV* gene product is pyrin, a protein thought to play an important role in the regulation of interleukin 1β (IL1β) and thereby of inflammation [3].

To date, according to Infevers, a database dedicated to auto-inflammatory mutations, more than 100 sequence variations have been identified in the *MEFV*, mostly due to single nucleotide substitutions [4]. Only a small number of these variants are unambiguously pathogenic and associated with the FMF phenotype. The most common pathogenic mutations worldwide are the p.M694V, M694I, M680I and V726A.

Although FMF was originally defined as an autosomal recessive disorder, approximately 25% of the patients carry only 1 *MEFV* mutation [5], and 10–20% carry no mutations at all [6]. Molecular analysis performed in previous studies confirmed the lack of other rare mutations in these patients [7,8]. Thus, the diagnosis of FMF still relies on clinical criteria [9], rather than genetic testing. Genetic analysis, if yielding, may help at most in atypical presentations [10].

The genetic flaw in the so called “FMF without *MEFV* mutations” is still elusive. The many theories exist may be condensed into two. One possible explanation is that this entity is associated with yet unknown upstream or downstream genetic defects in a component of the same metabolic pathway of pyrin. By this option genetic negative FMF patients are expected to bear a phenotype closely comparable to that of genetic positive FMF. Another possible explanation is that this subset of FMF is actually comprised of other autoinflammatory disorders, in which a thoroughly and meticulously characterized phenotype is expected to diverge from that of *MEFV* mutated FMF, even if initially appeared to agree with the clinical criteria of FMF. Thus, characterizing the phenotype of genetically “unaffected” FMF is essential for the understanding of the possible underlying genetic background of this entity.

Although mutation-negative FMF is part of many randomly studied cohorts of FMF patients [10-15], an extensive demographic and clinical characterization of this subset of FMF has never been the primary end point or the main focus of previous studies. The purpose of the current analysis is to thoroughly investigate this branch of FMF.

## Methods

All study and control patients, participating in the study were recruited from a cohort of FMF patients, coming to their regular visit, during 2012, at the Israeli national center for FMF in Sheba Medical Center, Tel-Hashomer and fulfilling the clinical criteria for diagnosis of FMF [9]. The patients and controls who consented, were recruited by one of us (CH), once monthly, at different weekdays and from different conducting physicians (the remaining authors), to increase random enrollment (several patient stocks) and uniformity of data collection (one compiler). Consecutive patients were enrolled successively, according to the order of arrival and divided into 3 groups, as follows:

- *Study group*: The study group consisted of *MEFV* mutation-negative FMF patients, identified as such according to their files. Patients were considered MEFV mutation negative, if underwent exon 10 (the most affected exon) sequencing and p.E148Q mutation analysis. This screening includes the 5 most common *MEFV* mutations in Israel (p.M694V, p.M694I, p.M680I, p.V726A and p.E148Q), and most *MEFV* rare mutations.
- *Control groups*: The control subjects comprised 2 FMF patient groups:
  1. Typical FMF population, consisting of genetically heterogeneous patients, with one or 2 mutations, excluding those who are mutation-negative. This reference group is important because it consists of a mixture of various phenotypes of FMF, giving the full spectrum of clinical FMF congruous with the definition of general FMF population.
  2. Most severe phenotype of FMF, consisting of FMF patients, homozygous for the p.M694V mutation. This group serves as a reference for the full blown form of FMF phenotype.

The 2 control populations were chosen as they represent both, the classical and the prototype (gold standard) phenotypes of FMF respectively. Placement of the *MEFV* mutation negative cohort phenotype within the boundaries outlined by the control groups increases the likelihood of its true association with the disease.

### Data collection and parameter definition

All study and control patients who signed an informed consent, were interviewed and their files were extensively reviewed. Based on these, a questionnaire detailing their clinical and demographic parameters was completed. Attacks of FMF were accounted for only if occurred repeatedly in the same site (>3 episodes) and were typical as described in the FMF criteria [9]. Triggers for attacks were those claimed by patients, based on their own experience, colchicine dose is the last recorded or declared. Intravenous colchicine was defined by administration of 1 mg of colchicine intravenously once weekly for at least 3 months. Proteinuria was defined as more than 300 mg/24 hrs, present at the time of the study or previously and lasting for at least 3 months, amyloidosis was diagnosed only if biopsy proven, chronic renal failure was defined by an elevated creatinine above normal limit (≥1.3 mg/dL) for at least 3 months, chronic arthritis was defined as arthritic episodes defined by a rheumatologist, lasting at least 1 month with or without radiographic changes. Splenomegaly was detected by physical examination or by imaging. Anemia of chronic disease was defined as hemoglobin lower than 11 g/dl (women) or 12 g/dl (men), with no other obvious cause. The Institutional review board of the Sheba Medical Center approved the study and all subjects signed an informed consent, according to the Declaration of Helsinki.

### *MEFV* mutation analysis

Mutation negative FMF patients were identified, based on file notes. In patients with limited genetic data, DNA was extracted from whole blood, using a commercial kit (high template polymerase chain reaction preparation kit; Roche Diagnostics, Indianapolis, IN). The p.E148Q was studied, using polymerase chain reaction and restriction enzyme analyses, as described elsewhere [16]. For exon 10 sequencing, the extracted DNA was amplified, using forward and reverse primers and fluorescent nucleotides, and sequencing was performed using ABI PRISM 3130 Genetic Analyzer (Applied Biosystems), as described elsewhere [17]. Of note, in our group, we relate to the p.E148Q variant as a mutation and not as a single nucleotide polymorphism (SNP), although with low penetrance, and usually mild phenotype. The *MEFV* mutation analysis adopted here covers around 96% of mutation carriage in the Israeli population [17].

### Severity scoring

The severity of FMF was determined using a scoring system developed by Mor et al. for FMF patients receiving colchicine [18]. Briefly, this score is comprised of the following parameters, each equals 1 point: Simultaneous involvement of more than one site during acute attack in at least 25% of attacks, more than 2 site of attacks during the disease course, colchicine dose of at least 2 mg/day needed to control the disease, 2 or more attacks of pleuritis during the disease course, 2 or more attacks of erysipelas like erythema (ELE) during the disease course and age of disease onset younger than 10 years old. A mild disease was defined by ≤ 1 point, moderate disease by 2 points and severe disease by ≥ 3 points.

### Statistical analysis

The Mann–Whitney U test was used for comparing nonparametric variables between the groups. For categorical parameters, the Fisher exact test was performed. All tests for significance were 2 tailed. P values < 0.05 were considered statistically significant.

## Results

Forty-seven *MEFV* genetic-negative, 60 genetically heterogeneous and 57 p.M694V homozygous FMF patients were enrolled. All fulfilled the criteria for diagnosis of FMF and all were recruited randomly and consecutively upon arrival to the clinic. The distribution of *MEFV* mutations in the genetically heterogeneous control group is shown in Table 1. The rate of patients with the homozygous p.M694V genotype in this group was approximately 33%, consistent with the frequency of this genotype in the Israeli FMF population [19].

**Table 1:** **Distribution of various MEFV genotypes in the genetically heterogeneous control group**

**Mutations**	**Rate N (%)**
p.M694V homozygous	20 (33.3%)
p.M694V heterozygous	10 (16.6%)
p.M694V compound heterozygous	16 (26.6%)
p.V726A Homozygous	3 (5%)
p.V726A Heterozygous	1(1.6%)
p.V726A/p.K695R	1 (1.6%)
p.V726A/p.E148Q	3 (5%)
p.E148Q Homozygous	2 (3.3%)
p.E148Q Heterozygous	4 (6.6%)

The demographic parameters are presented in Table 2. Compared with the 2 control groups, genetic-negative patients were significantly older at the onset of their disease and had significantly lower rate of patients with a positive family history for FMF. The severity of the disease, calculated according to the Mor severity score, was significantly milder in the mutation-negative patients, compared with the 2 mutation positive patient groups (Table 3).

**Table 2:** **Demographic parameters of MEFV mutation-negative FMF patients**

	**Mutation-negative (N = 47)**	**Heterogeneous controls (N = 60)**	**P value** ^*^	**p.M694V homozygous (N = 57)**	**P value** ^**#**^
Females (%)	31 (65.9%)	30 (50%)	0.117	36 (63.2%)	0.838
Age of disease onset (yrs)	19.61 ± 15.3	12.35 ± 10.36	**0.011**	5.76 ± 6.4	**0.0001**
Average diagnosis delay (yrs)	9.95 ± 11.2	6.91 ± 8	0.373	7.59 ± 9.5	0.234
Positive family history of FMF	21 (44%)	44 (73.3%)	**0.003**	45 (78.9%)	**0.0005**

*Mutation-negative vs. genetically heterogeneous controls.

^#^Mutation-negative vs. p.M694V homozygous.

Bold values=statistically significant.

**Table 3:** **Severity of FMF in MEFV mutation-negative patients**

**Severity score**	**Mutation-negative (N = 47)**	**Heterogeneous controls (N = 60)**	**P value** ^*^	**M694V homozygous (N = 57)**	**P value** ^**#**^
Mild	27 (57.4%)	23 (38.3%)		1 (1.8%)	
Moderate	9 (19%)	6 (10%)	**0.0049**	5 (8.8%)	**0.0001**
Severe	11 (23.4%)	31 (51.7%)		51(89.4%)	

*Mutation-negative vs. genetically heterogeneous controls.

^#^Mutation-negative vs. p.M694V homozygous patients.

Bold values=statistically significant.

Table 4 details the frequency of acute and chronic manifestations of FMF in all groups. Altogether, mutation-negative patients had a phenotype closely resembling the phenotype of the heterogeneous mutation-positive patients. Both groups had comparable rates of patients with acute abdominal, chest, skin and fever alone attacks, chronic FMF manifestations and intravenous colchicine treatment for refractory disease. The mean colchicine dose needed to control the disease was also similar between the groups. The only significant difference was a lower frequency of joint attacks in the genetic-negative group. None of the mutation-negative patients had amyloidosis or renal failure. Compared with the p.M694V homozygous patients, the mutation-negative cohort had a significantly lower rate of patients with acute FMF attacks in extra-abdominal sites, chronic arthritis, chronic renal failure, elevated parameters of chronic inflammation and the combined score of chronic manifestations of any type. In the mutation negative patient group, significantly lower doses of colchicine were required to control FMF attacks, compared with their homozygous p.M694V control peers.

**Table 4:** **Clinical characteristics of MEFV mutation-negative FMF patients**

**Parameter** ^**§**^	**Mutation-negative (N = 47)**	**Heterogeneous controls (N = 60)**	**P value** ^*^	**p.M694V homozygous (N = 57)**	**P value** ^**#**^
Average length of attack (days)	2.53 ± 1.36	2.86 ± 1.46	0.535	2.66 ± 1.5	0.857
Abdominal attacks	44 (93.6%)	53 (88.3%)	0.507	50 (87.7%)	0.509
Joint attacks	18 (38.2%)	37 (61.6%)	**0.020**	52 (91.3%)	**0.0001**
Chest attacks	18 (38.2%)	25 (41.6%)	0.842	36 (46.2%)	**0.017**
ELE	0 (0%)	5 (8.3%)	0.065	10 (17.5%)	**0.001**
Fever alone attacks	15 (31.9%)	12 (20%)	0.183	20 (35.1%)	0.835
Trigger for attacks	27 (57.4%)	34 (56.6%)	1.00	26/41 (63.4%)	0.663
Exertional leg-pain	25 (53.1%)	40 (66.6%)	0.168	47 (82.5%)	**0.002**
Average colchicine dose (mg/day)	1.54 ± 0.63	1.58 ± 0.53	0.727	1.9 ± 0.48	**0.003**
Intravenous colchicine treatment	2 (4.2%)	0	0.190	5 (8.8%)	0.452
Proteinuria above 300 mg/day	2 (4.2%)	1 (1.6%)	0.580	6 (10.5%)	0.288
Amyloidosis	0 (0%)	1 (1.6%)	1.00	5 (8.8%)	0.062
Chronic renal failure	0 (0%)	1 (1.6%)	1.00	6 (10.5%)	**0.031**
Kidney disease of any type^£^	2 (4.2%)	1 (1.6%)	1.00	6 (10.5%)	0.288
Chronic arthritis	2 (4.2%)	2 (3.3%)	1.00	11 (19.3%)	**0.034**
Splenomegaly	2 (4.2%)	3 (5%)	1.00	3 (5.3%)	1.000
Anemia of chronic disease	4 (8.5%)	8/43 (18.6%)	0.217	3 (5.3%)	0.698
Chronic inflammation (SAA > 10 mg/l)	3 (6.3%)	11 (18.3%)	0.086	14/53 (26.4%)	**0.008**
Combined chronic disease features&	26 (55.3%)	44 (73.3%)	0.06	48 (84.2%)	**0.0021**

ELE- Erysipelas like erythema, SAA- Serum amyloid A.

^§^See definitions of parameters in the method section. * Mutation-negative vs. unselected controls. ^#^ Mutation-negative vs. p.M694V homozygous. ^£^ Including proteinuria above 300 mg/day, amyloidosis and chronic renal failure. & including exertional leg-pain, proteinuria above 300 mg/day, amyloidosis, chronic renal failure, chronic arthritis, splenomegaly and anemia of chronic disease. Bold values=statistically significant.

## Discussion

Our findings suggest that mutation-negative FMF patients display a phenotype only slightly different from that of heterogeneous mutation positive FMF patients, seen in a large FMF clinic in Israel. Compared to the most severe phenotype of FMF (patients who are homozygous for the p.M694V *MEFV* mutation), mutation-negative patients present a quantitative difference, expressed mainly in disease severity, which was much milder with regard to the acute and chronic manifestations and the colchicine dose needed to control their attacks.

*MEFV* mutation-negative FMF has never been in the focus of a study. A limited description of their phenotype however, can be occasionally encountered in a small number of studies, in which this subgroup emerged inadvertently as a separate group. Padeh et al. and Ozturk et al. described the phenotype of mutation negative pediatric patients as part of a broader genetic study of FMF [10,14]. Their findings are in line with ours in most parameters, including predominance of abdominal attacks and lower rates of attacks at other sites. In Ozturk’s study, also in line with our results, *MEFV* mutation negative FMF patients had lower frequency of a family history of FMF and their clinical phenotype was comparable to patients who have 1 or 2 *MEFV* mutations. Contrary to our findings however, approximately 5% of the mutation-negative patients in Ozturk’s study developed amyloidosis. This difference could be explained by different trends in patient management between the countries and other environmental factors affecting the propensity to develop amyloidosis [20].

Two methodological notes should be mentioned. First, an extensive search for a second mutation in heterozygous patients, including entire *MEFV* genomic region sequence, did not identify one [7,8]. These findings support the accuracy of our screening methodology of *MEFV* genetic analysis to detect mutation negative FMF patients, and confirm with high certainty that our cohort is a true subset of patients with unaffected *MEFV*, and not a group of patients in whom *MEFV* mutations have been missed. Secondly, our genetically heterogeneous control group represents the typical phenotype of FMF, as reported in various cohorts around the world, with regard to disease severity, manifestations of attacks, family history of FMF and colchicine treatment [15,21,22]. The frequency of different *MEFV* mutations is also comparable to that of the general FMF population seen in Israel [19]. Thus, it appears that the heterogenous control patients enrolled in the present study may serve as an appropriate reference for the “common” FMF phenotype, and used for comparison with the subset of interest in this study, the mutation-negative patients.

The current study on *MEFV* mutation negative FMF brings us to at least 3 comprehensions. One regards the genetics of FMF, the second related to the phenotype of FMF and the third to the origin of FMF without *MEFV* mutations. As for the genetics, it has been already shown that the phenotype expressed by FMF patients, carrying a single *MEFV* mutation, is only slightly different from that of patients with 2 *MEFV* mutations [21,23]. Our study further extends this finding, showing that the same is true for patients who carry no *MEFV* mutations at all. Altogether, these findings underline the notion that FMF, as currently perceived, is a clinical entity with complex genetic background and it cannot be diagnosed or excluded based on *MEFV* genotyping alone, giving an extra weight for the need of a specific laboratory test to distinguish FMF from other forms of autoinflammatory diseases. Until such a measure is available, the merit of stringent criteria (for instance peritonitis vs. just abdominal pain or mono-arthritis of lower extremities vs. just arthritis) is obvious and should be strived for.

Regarding the phenotype, compared with the regular phenotype of FMF, *MEFV* mutation-negative patients have a late disease onset, milder disease severity, and lower rate of positive family history for FMF. These characteristics put mutation-negative patients at the milder end of the disease spectrum, together with other distinct groups of FMF patients with milder disease severity, including Ashkenazi and Iraqi Jews [24,25], patients with late onset of their disease [26] and patients who experience a colchicine-free remission [27]. In general, these subsets of FMF patients have lower rates of extra-abdominal attacks, ELE, chronic arthritis, chronic manifestations including chronic kidney disease and amyloidosis and lower rate of elevated markers of chronic inflammation [28]. In contrast, in different cohorts, including ours, these patients have a relatively high rate of typical abdominal attacks (in over 85% of patients), just like patients with the severe form of FMF (p.M694V homozygous patients). Thus, it seems that typical abdominal attacks dominate the clinical picture and is the single most sensitive manifestation of FMF, found across all patient subgroups, including those with milder disease, as is the case for the study group in the present work.

Finally, in relation to the root of FMF without MEFV mutations, it is unclear, and there is a myriad of hypotheses, including misdiagnosis of other autoinflammatory disorders as FMF, epigenetic changes such as DNA methylation or hisotne modifications of *MEFV*, an interplay between genetic polymorphisms, modifier genes and environmental factors culminating in FMF spells, and mutations in yet unknown different genes causing a similar disease. Another possible recent explanation for *MEFV* mutation-negative FMF might be somatic mosaicism, a phenomenon discovered lately, to underly NLRP3 genetic-negative cryopyrin-associated periodic syndrome, a genetically different autoinflammatory entity [29,30]. In general however, these possibilities might be narrowed to 2: defects in *MEFV* associated pathway or another autoinflammatory disease. Our findings showing close resemblance of mutation negative FMF to mutation positive FMF supports the former paradigm.

## Conclusions

In conclusion, *MEFV* mutation-negative FMF presents a phenotype only slightly distinguishable from that presented by FMF patients with 1 or 2 *MEFV* mutations, generally suggesting a shared metabolic pathway with genetically positive FMF. Better understanding of the genetics and the pathogenesis of this subset of FMF is a target for future studies.
